# Surrogate virus neutralization test to determine salivary neutralizing antibody inhibition of ACE2 and SARS-CoV-2 spike RBD complex

**DOI:** 10.3389/fimmu.2026.1803393

**Published:** 2026-06-18

**Authors:** David Forsman, Abigail Smith, Alex Pratt, Paige Comerford, Fiona Sparano, Congyue Peng, Delphine Dean

**Affiliations:** 1 Department of Bioengineering, Clemson University, Clemson, SC, United States; 2 Department of Biological Science, Clemson University, Clemson, SC, United States; 3 Research and Education in Disease Diagnostics and Intervention, Clemson University, Clemson, SC, United States

**Keywords:** neutralizing antibody, receptor binding domain (RBD), saliva, SARS-CoV-2, sVNT

## Abstract

The viral pathogen SARS-CoV-2 caused a pandemic with detrimental effects across multiple domains. Mutations that gain transmission and infectability advantages have complicated virus prevention and mitigation. SARS-CoV-2 virus gains entry to human cells through the complex formed between the human Angiotensin Converting Enzyme 2 (hACE-2) and the viral spike protein. Neutralizing antibodies can block the binding of the spike protein to hACE-2, thereby neutralizing the virus. These antibodies are present in mucosal fluids and plasma. In this study, we demonstrate the deployment of a laboratory-developed surrogate virus neutralization test (sVNT) to assess the neutralizing activity of antibodies in saliva. Samples are collected from donors who completed the first two doses of the Pfizer vaccine, on average 36.6 weeks apart, and received the booster shot in September 2021. We analyzed the neutralizing activity of the saliva antibody against the spike receptor-binding domain (RBD) of the reference spike and the Omicron variant. The analytical sensitivity and specificity of the laboratory-developed sVNT are comparable to those of the FDA-approved sVNT. Vaccine-elicited saliva antibodies were able to block the binding of hACE-2 to the spike RBD of the reference strain. However, Omicron spike RBD abolished saliva neutralizing ability, implicating escape from mucosal immune defense. The experience we gained from assessing SARS-CoV-2 virus neutralization in saliva can be broadly applied to urgent needs against other pathogens, enabling us to quickly deploy a surrogate neutralization test to evaluate immune protection.

## Introduction

The severe acute respiratory syndrome coronavirus 2, or SARS-CoV-2 infection has been a common point in everyone’s lives since 2020. Between changes in lifestyle, relationships, and, most certainly, health, the world is a much different place than it was just a few years ago. COVID has led to the infection and the death of more than seven million people (World Health Organization 2026 data). SARS-CoV-2 infection has symptoms like shortness of breath, dry cough, fatigue, and headaches (1). An infection from SARS-CoV-2 can lead to endothelial damage, thrombosis, and inflammation (2). The viral particle infects humans by binding to human cell receptor ACE-2 (hACE-2) through hydrogen bonding and hydrophobic interaction of the amino acid side chain (3). The host also produces CD4+ and CD8+ lymphocytes at the time of the first exposure (4). When re-exposed to viral antigens, these cells multiply and mature into effector cells more rapidly than during a primary immune response (5).

Salivary antibodies include the secretory IgA (S-IgA), secretory IgM (S-IgM), and IgG. S-IgA and S-IgM are produced by local plasma cells that are adjacent to the ducts and acini of salivary glands. Salivary IgG is passively leaked from the blood circulation through gingival crevicular epithelium. IgG antibodies are found present in mucosal fluids and plasma (6). Peak IgG was found in saliva samples about 16–30 days after the initial COVID symptom onset (7). A persistent amount of IgG can be detected after 12 months in saliva post-COVID infection (8). Virus infection and therapeutic interventions such as vaccines can elicit antibodies of different isoforms and at variable titers. The mucosal immune response plays a vital role in preventing viral reinfection (9). Most viruses infect the body through mucosal portals (10). Mucosal tissues contain antiviral antibodies and CD8+ lymphocytes that can rapidly identify and neutralize specific viral antigens (11). Mucosal plasma cells secrete IgA antibodies, which are transported to mucosal surfaces to support mucosal immune defenses (6). Previous studies indicate that the mRNA BNT162b2 vaccination against the reference SARS-CoV-2 strain elicits a strong systemic immune response by significantly increasing the concentration of neutralizing antibodies in blood serum (12). However, the vaccine did not significantly increase neutralizing antibodies in the saliva. Oral mucosal immunity responded poorly to the vaccine and failed to neutralize the virus via this route (13).

Neutralizing antibodies (nAbs) are the primary defense against infection in the body (14). They are a subset of antibodies that bind near or at the active site and neutralize the functional activity of the virus particle. The accuracy of assays for detecting total antibody (IgA and IgG) has been examined (15), but little work has been done on the assay performance in neutralization tests specifically using surrogate viruses. nAbs have been at the center of SARS-CoV-2 treatment (16) and are the backbone of defense against the virus, blocking viral entry, recruiting natural killer cells, and facilitating phagocytosis (17). The Omicron variant of the virus has multiple mutations compared to previous strains, including 15 in the receptor-binding domain (RBD) (18). Of these 15 mutations in the RBD, two play key roles in immune escape and viral infectivity (18). We expect that the neutralizing antibodies elicited by the Pfizer-BioNTech booster dose may have lower neutralization efficacy against the Omicron variant of the spike RBD. Previously, we employed microfluidic diffusional sizing (MDS) to measure the binding affinity of the salivary nAb against SARS-CoV-2 spike RBD (19). In this study, we developed a laboratory-derived semi-quantitative saliva neutralization test that is analogous to the commercially available option (cPass™ Surrogate Virus Neutralization Test (sVNT) (Genscript)). The Neutralization Assay tests the extent of inhibition the antibody exerts on the binding of the virus receptor-binding domain to the cell receptor. Using this Laboratory developed test (LDT) neutralization test, we assessed the level of inhibition and neutralization of the spike RBD in saliva samples collected from a cohort participant receiving the Pfizer booster vaccination. We examined whether the saliva antibody elicited by the booster shot can neutralize both the original and the Omicron version of the spike RBD.

## Materials and methods

### Pooled saliva

Pooled pre-COVID saliva was purchased from Lee Biosolutions (Item # 991-05-P-PreC-250).

### Study cohort

The study group included 20 participants who were getting the Pfizer booster mRNA shot. Participants voluntarily provide paired saliva samples at the time the shot is administered (designated as Week 0) and each week thereafter for up to 10 weeks. The participants have no history of Omicron infection. One of the saliva samples from each collection was subject to SARS-CoV-2 RT-qPCR antigen test (20). The second saliva sample was tested for neutralization of the original and Omicron spike RBDs. The collection follows the approved Institutional Review Board (IRB) protocol (IRB # Pro00100731) by the Clemson University IRB committee with signed donor consent.

### Recombinant spike RBD domain (original), spike RBD (Omicron), and the cPass sVNT kit

The horseradish peroxidase (HRP) labeled recombinant spike RBD domain (wuhan-1 reference, accession # NC 045512), the HRP labeled recombinant spike RBD (Omicron), human ACE2 (hACE2), and the cPass SARS-CoV-2 Surrogate Virus Neutralization Test kit were purchased from GenScript Inc.

### sVNT

The laboratory-derived sVNT for salivary neutralizing antibody assay is developed based on the cPass SARS-CoV-2 Surrogate Virus Neutralization Test kit (Genscript). Hi-bind polyethylene plates were coated with hACE 2 (at 15 µg/mL). The uncoated area was blocked with a 5% milk solution and then washed with 200 μL of 1x PBST three times. An HRP-RBD solution was prepared by diluting HRP-conjugated RBD (GenScript Z03594) 1:1000 in 1x PBST supplemented with 1% milk. Recombinant monoclonal antibody (PDB #7SBU, J08 Fab) that is shown neutralization to the spike RBD is purchased from Excelgene (mAb-61 (XLG-SARSCoV2-mAb-3). A serial concentration of 0 U/mL, 3.125 U/mL, 6.25 U/mL, 12.5 U/mL, 25 U/mL, 50 U/mL, 100 U/mL, and 200 U/mL was made to generate a standard curve for each plate. For analytical specificity and sensitivity, randomized pre-COVID saliva with or without the addition of the anti-spike RBD nAb (GenScript A02087) was used. For clinical specificity and sensitivity, saliva samples collected from the donors were used. Saliva samples were centrifuged at 2600 x *g* for 15 minutes (21), and the supernatant was collected and incubated with 1% Triton X-100 for 1 hour at room temperature. Triton X-100 treated saliva samples were then 1:5 diluted by 1x PBS supplemented with 1% milk and mixed with an equal volume of the HRP labeled spike RBD and incubated at 37 °C for 30 minutes. After incubation, the mixture was added to the hACE2-coated plate in duplicate and incubated for 15 minutes at 37 °C. The plate was washed with 200 μL of 1x PBST three times. 100 μL of TMB (3,3’, 5,5;-tetramethylbenzidine) solution was added to each well and left to incubate for 15 minutes in a dark space. 50 μL of 0.32 N sulfuric acid stop solution was added to each well, and the absorbance was recorded at 450 nm by a BioTek spectrometer. The results from the LDT neutralization were compared with the results from the class SARS-CoV-2 sVNT (Genscript, L00847-A), which was performed according to the manufacturer’s instructions.

### ELISA

Hi-bind polyethylene plates were coated with spike RBD at 2 μg/ml overnight and stored at 4 °C. The uncoated area was blocked with 250 µl 5% milk in 1x PBST and then washed with 300 μL of 1x PBST for three times. Saliva samples were 1:5 diluted using 1x PBST supplemented with 5% milk. 100 μL of prediluted saliva was added, and the mixture was incubated at room temperature for 1 hour. Wash the plate with 1x PBST three times. 100 μL of HRP-conjugated anti-IgG (Invitrogen, 1:4000 diluted in 1x PBST with the supplement of 5% milk) was added. The plate was left at room temperature for 1 hour with constant shaking. Wash the plate three times with 1x PBST, then add 100 μL of TMB solution to each well and incubate for 15 minutes in the dark. 50 μL of 0.32 N sulfuric acid stop solution was added to each well. The reaction was stopped by 100 μL of 0.32 N sulfuric acid. The absorbance was recorded at 450 nm by a BioTek spectrometer.

Data analysis and visualization were performed using JMP Pro 16 and MATLAB (R2023a).

## Results

### Detection limit and analytical specificity and sensitivity

**Figure 1:**
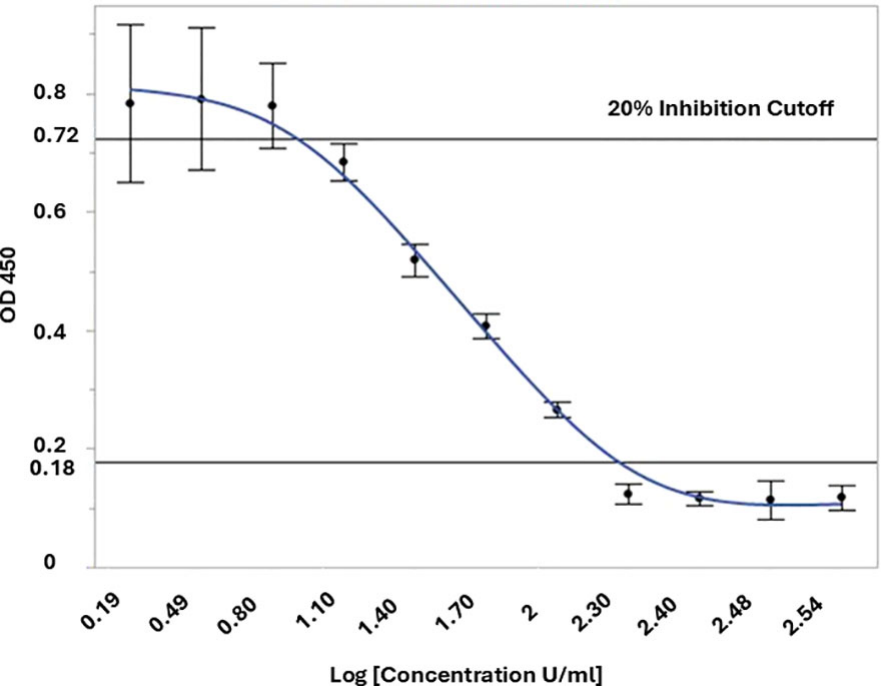
Detection limit and standard curve of the laboratory-derived surrogate neutralization test (sVNT). Antibodies (recombinant anti-spike IgG derived from PDB #7SBU, J08 Fab)) were purchased from ExelGene and serially diluted with 1x PBS. The LLOQ was determined by graphing % Inhibition vs. OD 450 and interpolating the value at 20%. The ULOQ was determined by multiplying the standard deviation by 3 and adding it to its corresponding OD 450 value. These values were then averaged to determine the ULOQ. Each error bar indicates ± standard deviation (STD DEV).

Under the conditions we tested, using 15 µg/mL hACE2 coated plate, a standard curve was generated using different concentrations of a recombinant anti-spike monoclonal antibody standard (Excelgene XLG-SARSCoV2-mAb-3) known to neutralize the spike protein. The concentration of the antibody standard is plotted on a logarithmic scale (**Figure 1**). The upper limit of quantification (indicated by the black line at the bottom of the graph) is determined as the average of the OD 450 values for the top three highest antibody standard concentrations plus 3 times their standard deviation. Based on the linear regression of plotting the OD 450 absorption vs. the percentage inhibition, the lower limit of quantification (indicated as the black line at the top of the graph) is determined from the 20% inhibition of the binding of hACE2 and spike RBD.

Analytical specificity and sensitivity were determined using pooled pre-COVID and pooled pre-COVID contrived with the recombinant antibody standard. In this part of the assessment, the test samples were prepared as follows: negative samples were prepared using negative saliva alone; a positive sample was prepared using negative saliva spiked with a known concentration of a neutralizing antibody standard. A total of 100 test samples were prepared for the assessment of analytical specificity and sensitivity. The randomized pooled pre-COVID with or without the addition of the antibody standard (2U/µl) was tested using laboratory-derived sVNT (a total of 100 tests) and compared with the FDA-approved cPass sVNT (a total of 120 tests). Analytical specificity was calculated as the percentage of true negatives among the total number of false positives and true negatives. Analytical sensitivity was calculated as the percentage of true positives relative to the total number of true positives and false negatives. We estimated that LDT sVNT has a 100% analytical specificity, and so does the commercialized assay (cPass sVNT kit). The analytical sensitivity of the LDT is 77.4 ± 1.96%, compared to 80.6 ± 7.1% for the commercialized assay (**Table 1**). There is no significant difference in analytical sensitivity between the LDT and the commercial kit. Intra- and inter- assay coefficient of variation (*%CV*) was 7.85 and 9.67, respectively for the In-house plate using the contrived saliva samples, i.e. the negative saliva spiked with nAb standards (**Supplementary Table 1**). The intra- and inter-assay coefficient of variation (*%CV*) was 1.88 and 9.96 for IgG, respectively, and 2.10 and 33.08 for IgA, respectively, using the contrived saliva samples, i.e. the negative saliva spiked with IgG or IgA standards (**Supplementary Table 1**).

**Table 1:** Summary of the analytic sensitivity and specificity of the laboratory developed (in house) assay and the commercial sVNT assay.

Commercial Assay Analytical Criteria			Total	In House Assay Analytical Criteria			Total	Legend:	Positive	Negative	Total
	Positive	Negative			Positive	Negative					
Positive	25	0	25	Positive	24	0	24	Positive	# of True Positive (TP)	# of False Positive (FP)	TP+FP
Negative	6	29	35	Negative	7	19	26	Negative	# of False Negative (FN)	# of True Negative (TN)	FN+TN
Total	31	29	120	Total	31	19	100	Total	TP+FN	FP+TN	N
								# of Analytical Positives:	31	# of Analytical Negatives:	29
Analytical Estimated Sensitivity (sens):		80.6 +/- 7.1		Analytical Estimated Sensitivity (sens):		77.4 +/- 1.96		Clinical Estimated Sensitivity (sens):		sens = 100 x [TP / (TP +FN)]	
Analytical Estimated Specificity (spec):		100.0		Analytical Estimated Specificity (spec):		100.0		Clinical Estiamted Specificity (spec):		spec = 100 x [TN /(FP+TN)]	
Based on evaluation specimen with disease prevalence:		25.8		Based on evaluation specimen with disease prevalence:		31.0		Based on evaluation specimen with disease prevalence:		100 x [(TP +FN)/N]	
Study Predicitve Value of Positive Test Result (PPV):		100.0		Study Predicitve Value of Positive Test Result (PPV):		100.0		Study Predicitve Value of Positive Test Result (PPV):		PPV = 100 x [TP/(TP + FP)]	
Study Predicitve Value of Negative Test Result (NPV):		82.9		Study Predicitve Value of Negative Test Result (NPV):		73.1		Study Predicitve Value of Negative Test Result (NPV):		NPV = 100 x [TN/(TN + FN)]	

### Clinical specificity and sensitivity

Neutralization of viruses in the mucosal interface has been recognized as a critical defense mechanism against virus infection (22). The weekly changes of the nAb detected by both the commercial GenScript sVNT and the In-house sVNT were graphed in **Supplementary Figures 1**, **2**. A corresponding bar graph is also shown to demonstrate the overall nAb fluctuation by week. The IgG and IgA titer of the respective patients are also graphed, shown in **Supplementary Figures 3**, **4**. Clinical specificity was calculated as the percentage of true negatives among the total number of false positives and true negatives. Clinical sensitivity was calculated as the percentage of true positives among all true positives and false negatives. 64 clinical saliva samples from the participant cohort (**Table 2**) were tested. The neutralizing inhibition of ACE2 binding to SARS-CoV-2 spike RBD domain was steadily detected in saliva samples using both the commercialized semi-quantitative RUO kit and the LDT (N = 64 **Table 3**). The LDT has 100% clinical specificity and 56.5 ± 10.3% clinical sensitivity. We used the same amount of hACE2 to coat the plate as we used in assessing the analytical specificity and sensitivity. Because the assay calculates the percentage of inhibition of binding between hACE2 and the spike RBD, the clinical sensitivity of the LDT assay depends on the amount of hACE2 used to coat the plate. Our data suggests that the ample amount of hACE2 used for competitive binding can be tested to determine the optimal clinical sensitivity for each sample type and the range of nAb present in the sample.

**Table 2:** Summary of the demographics of the participants.

Sample Size	Age (Mean)	Gender	Average number of weeks between second vaccine shot and the booster
Patient (N=20)	50	Female (85%)	45
Samples (N=9)		Male (15%)	

**Table 3:** Summary of the clinical sensitivity and specificity of the laboratory-developed (in house) sVNT assay compared against the reference commercial GenScript sVNT assay.

Clinical Criteria				Total
		In- House		
		Positive	Negative	
Commercial	Positive	13	0	13
	Negative	10	9	19
	Total	23	9	64
Clinical Estimated Sensitivity (sens):			56.5 +/- 10.3	
Clinical Estiamted Specificity (spec):			100.0	
Based on evaluation specimen with disease prevalence:			35.9	
Study Predicitve Value of Positive Test Result (PPV):			100.0	
Study Predicitve Value of Negative Test Result (NPV):			47.4	

### Salivary spike RBD neutralization response to canonical spike RBD and the Omicron spike RBD after the BioNTech & Pfizer first booster shot

**Figure 2:**
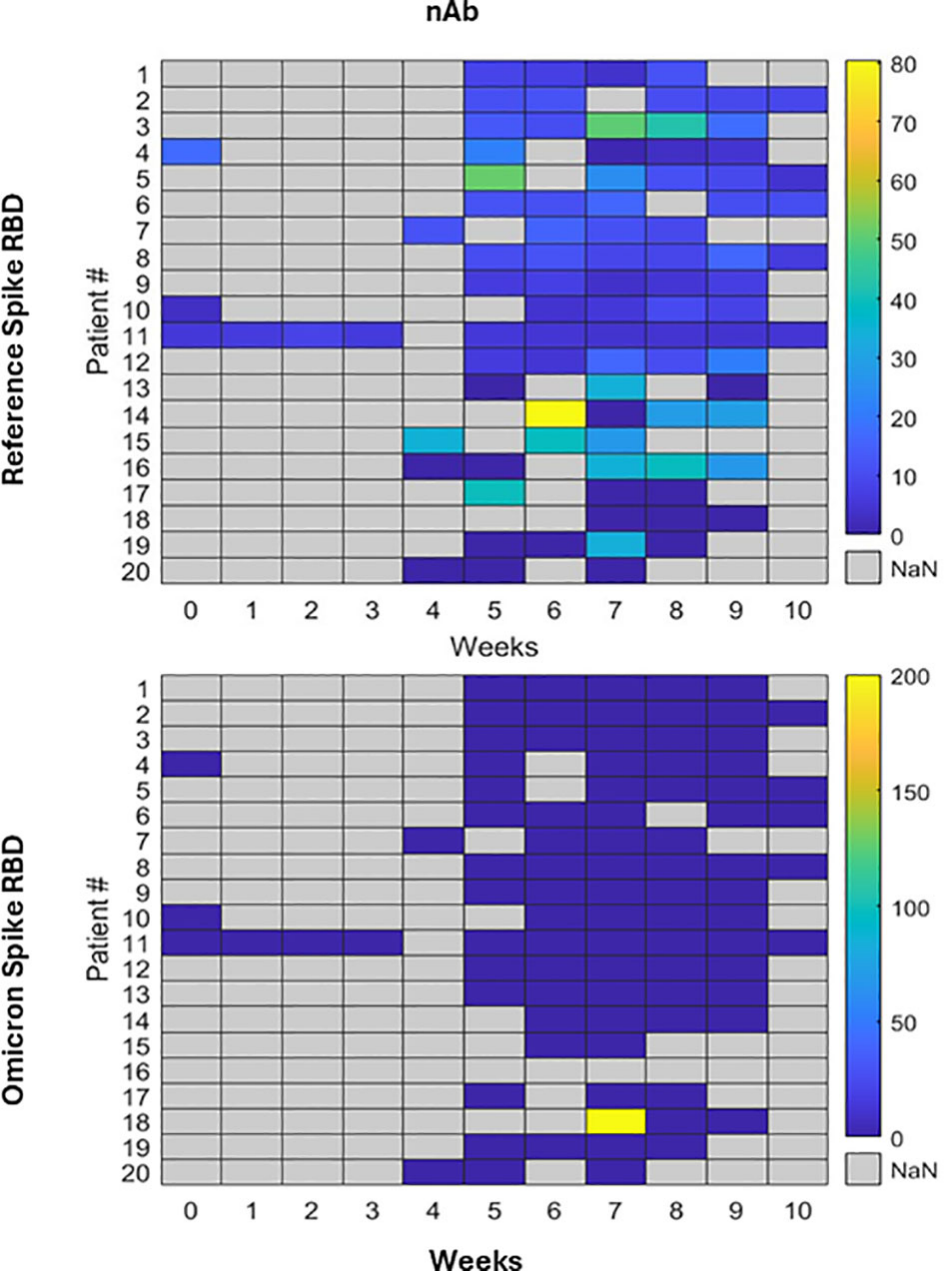
Heatmap of the detected nAb titer against reference spike RBD and the Omicron spike RBD from the saliva samples provided by the study participant at different weeks post the booster Pfizer mRNA vaccine. The color gradient (from blue to yellow) is a quantitative indicator of the antibody titer in the samples (low to high). The grey slots indicate no samples were received from the patient for that week.

**Figure 3:**
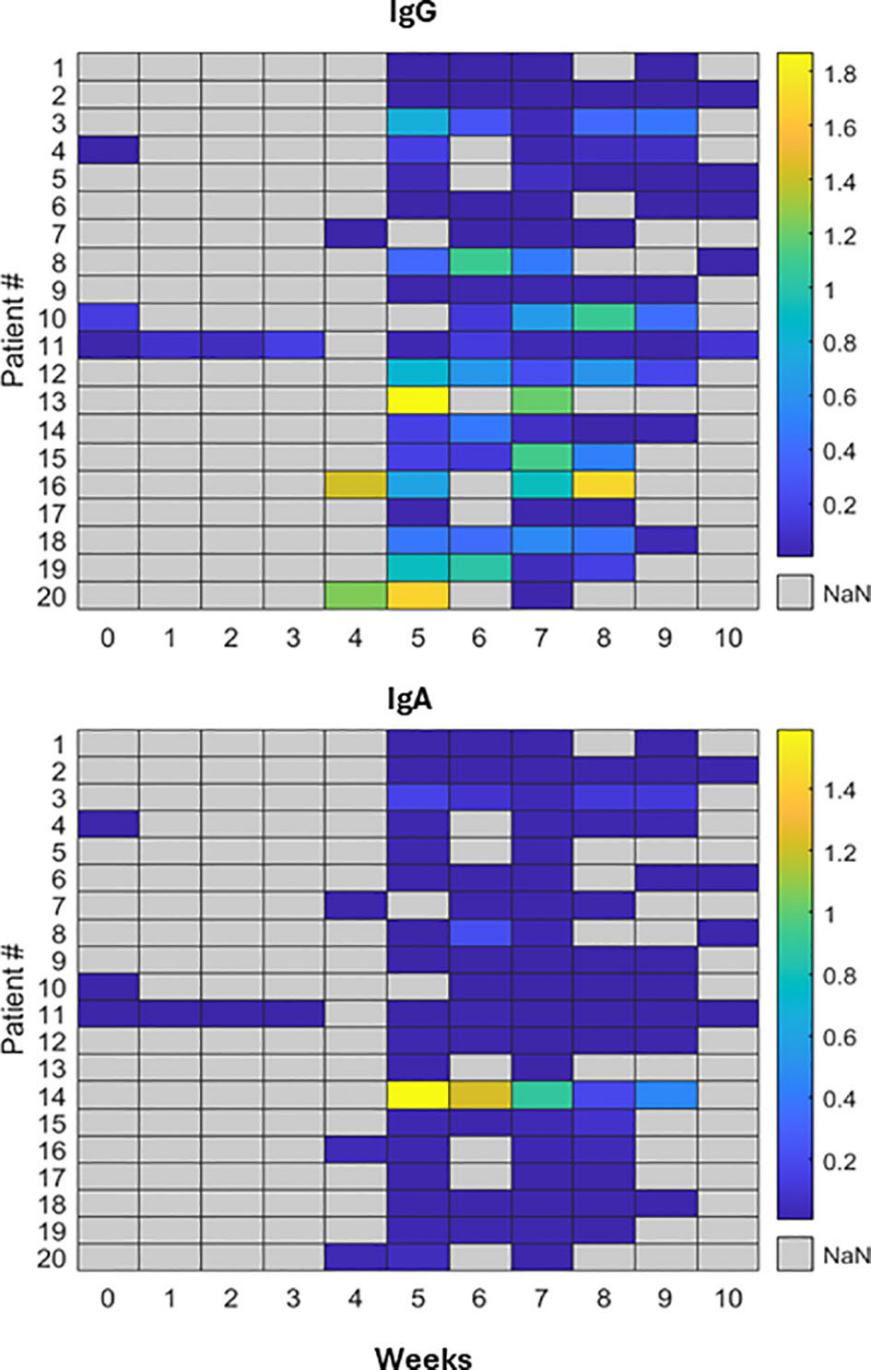
Heatmap of the total IgG and IgA antibody titer against reference spike RBD from the saliva samples provided by the study participant at different weeks post-booster Pfizer mRNA vaccine. The color gradient (from blue to yellow) is a quantitative indicator of the antibody titer in the samples (low to high). The grey slots indicate no samples were received from the patient for that week.

**Figure 4:**
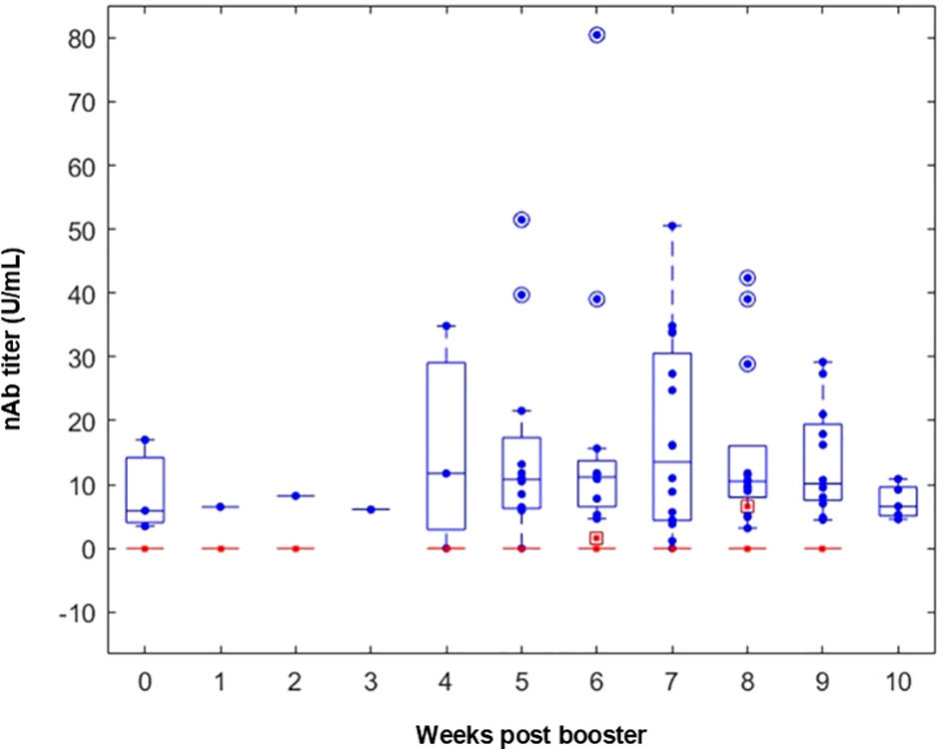
Comparison of neutralization antibody titer against the Spike RBD (original version, indicated in blue) and the Spike RBD (Omicron version, indicated in red) from the saliva samples provided by the study participant at different weeks post-booster Pfizer mRNA vaccine.

After the clinical sensitivity and specificity of the semi-quantitative LDT assay were evaluated, we estimated the nAb titer in saliva samples collected from participants who received the Pfizer mRNA booster shot, which was deployed at the end of September 2021. This is the first booster shot released after the two doses of the original shot. The cohort information is listed in **Table 2**. A total of 20 participants were enrolled in the self-collection of duplicated saliva samples from the day the booster shot was administered for a consecutive period of 10 weeks. A total of 92 weekly saliva samples were received. One of the duplicated samples was sent for the saliva clinical SARS-CoV-2 positivity test (20). All samples returned negative SARS-CoV-2 RT-qPCR test results within the time period during which the samples were collected. The duplicate was tested for salivary virus neutralization using the LDT sVNT. The neutralizing antibody titer against the reference spike RBD (NC_045512) is indicated as a color gradient for each participant each week (**Figure 2**). Grey indicates that no samples were received from participants that week. Due to the chaotic situation during the Omicron surge during our sample collection period, only a handful of samples were received from weeks 0-3. Samples were mostly collected in week five, and we observed low to modest neutralizing antibody titers against the canonical spike RBD in the received samples. There is individual variation in the salivary neutralizing antibody being detected. Total IgG and IgA amounts were estimated using the ELISA. We detect a detectable amount of IgG across the collection period, but IgA is barely detectable in most samples (**Figure 3**). When these samples were tested against the Omicron spike RBD, we observed a significant reduction in salivary neutralization. When plotted against the weekly neutralizing antibody titer against the canonical spike RBD, we show that the amount of inhibition of the salivary neutralizing antibody against the Omicron spike protein is minimal compared to the inhibition against the canonical spike RBD (**Figure 4**). We show that the Omicron spike RBD escapes salivary antibody neutralization in individuals up to 10 weeks after the first Pfizer booster shot. Saliva samples from subjects showed steady levels of nAbs, with detectable total IgG antibodies against the reference spike, up to 10 weeks post-booster vaccination, as determined by ELISA (**Figure 3**). The total IgA antibodies against the reference spike RBD are barely detectable in most of the samples received. The total IgG and IgA against Omicron spike RBD were undetectable in the samples collected. This data indicates that the salivary neutralization test can be used to assess the oral mucosal immunity against SARS-CoV-2 reference strain and the variant. Inhibition by neutralizing antibodies of the formation of the ACE-2 and spike RBD complex is significantly higher for samples incubated with the reference spike RBD than for samples incubated with the Omicron RBD.

## Discussion

### sVNT assessing salivary immune response

Neutralizing antibody detection can occur through multiple routes. Live-viruses and cell-based assays are the gold standard. However, only a handful of labs equipped with live-virus-handling facilities can perform those assays. We previously developed a microfluidic-based neutralization test (19). Mancuso and the group used the commercially available sVNT (Genscript) to evaluate neutralizing antibody levels in sera after BNT162b2 vaccination (23). The commercial sVNT (Genscript) is 89.8% sensitive and 100% specific in the clinical sera sample compared to the plaque-reduction neutralization test (24). Here, we show the development of an LDT sVNT. The purpose of developing an In-house assay is to provide an example of an sVNT developed in the lab for saliva samples. For rural areas that less resources are allocated, a reliable In-house assay can avoid the reliance on commercial accessibility, and allow quick modification as the analytical target mutates. We demonstrate the procedure for developing and validating an sVNT assay in the lab, which is particularly useful to evaluate saliva virus neutralization when commercial assays are not readily accessible. The advantages of the surrogate virus tests are that they are easy to apply and relatively inexpensive. Because the sVNT assay calculates the percentage of inhibition of hACE2 binding to the spike RBD, the assay’s limit of detection and sensitivity depend on the amount of hACE2 coated on the plate. The assay can be designed by empirically adjusting the hACE2 amount to balance assay sensitivity and meet the detection needs at higher levels. We also suggest running tests on specific sample types to identify the parameters best suited to each sample. The sVNT results in this study showed us that Omicron neutralization is completely abolished in saliva when directly compared with the reference spike RBD (accession # NC_045512). The saliva sample showed significantly greater neutralization against the reference spike RBD. It is inferred that the lack of neutralization of the Omicron spike RBD is due to altered antibody specificity caused by the mutation; however, we don’t have direct proof of whether this is due to mucosal antibody titers or altered antibody specificity. sVNT is an inhibition assay; its nature limits its ability to demonstrate the mucosal antibody titer.

We observed high individual variability (outliers) in the sVNT, IgG, and IgA assays. We performed the Spearman correlation analysis of the nAb and the sum of the IgG and IgA. We found no monotonic correlation (*ρ* = -0.123) between nAb and the total IgG and IgA titer. Whereas a weak monotonic correlation (*ρ* = 0.285) is found between IgG and IgA (**Supplementary Table 2**). This is not surprising considering that the IgA and IgG assays were performed after we optimized and validated the sVNT test. We speculate that an unproportional decay of IgG and IgA may contribute to the weak correlation. In addition, the sVNT in the study evaluates total neutralization, including antibodies, but may also include non-antibody molecules that inhibit the binding of the spike RBD to hACE2 (25). Neutralization antibody titers may be overestimated for this reason. Additional tests, such as G and A protein beads that can bind to the antibodies, may be added before the test to verify the presence of the antibody. A mass spectrometry test can also be performed to determine the antibody sequence. Mucosal neutralization antibodies are understudied. Their sequence in the database is scarce. Further determination of the neutralization antibody sequence in saliva will provide guidance for developing antibody-based therapeutic strategies.

The LDT sVNT developed in this study has 100% analytical and clinical specificity. This suggests that this LDT can reliably evaluate the neutralization inhibition of samples against the binding complex of hACE2 and the spike RBD. The analytical sensitivity of the assay is 77.4 ± 1.96%. Using this special cohort and the specific assay settings described in the method section, we assessed clinical sensitivity at 56.5 ± 10.3%. For this inhibition assay, the assay performance is directly related to the amount of neutralizing antibodies in the sample relative to the amount of hACE2 coated on the plate. When a smaller amount of hACE2 is coated on the assay plate, the inhibition can be detected with less neutralizing antibody presence, therefore, a higher sensitivity. But it will be saturated easily if the amount of neutralizing antibody exceeds the linear range. To tailor this test to evaluate the neutralizing antibody response against other microorganisms, it is beneficial to gauge the amount of antigen coated on the assay plate to achieve an acceptable sensitivity without compromising the maximal detection range. This assay can be quickly deployed once the target receptor is identified. It provides an alternative solution to the survey neutralization capability in a large population, fast and cost-effective.

### Neutralizing antibody in saliva

Mucosal immune defense is a critical yet neglected step to prevent SARS-CoV-2 infection (26). Mucosal neutralization in the lower respiratory tract against the Omicron variant is significantly lower than in paired plasma samples from children with severe COVID symptoms (27). We detected salivary IgG and IgA, as did other studies that reported salivary and nasal IgG and IgA antibody levels (28, 29). Although IgA antibody levels are reduced after 10 weeks of symptom onset (30, 31), total anti-spike RBD IgG and IgA antibodies were detected in saliva samples after the first and second doses of the initial Pfizer SARs-CoV-2 mRNA administration (32). Detection of IgA in saliva may only be limited to the protease-susceptible IgA subtype, IgA1 (33). But the salivary IgA and IgG were not significantly increased after the Pfizer booster dose (34).

Using the Focus Reduction Neutralization assay (FRNT) and live SARS-CoV-2 viruses, Nickel *et al.* (35) determined that neutralization antibody titer against the reference spike RBD is detectable in serum in the patients post-BNT162b2 booster, and there is a reduced amount of neutralization antibody titer detected against the Omicron spike RBD. However, neutralizing antibodies against the reference spike RBD are significantly reduced in sera after the second dose of the Pfizer-BioNTech BNT162b2 vaccine (36).

Garziano and group used a live-virus and cell assay to confirm that the saliva sample’s neutralization of the reference spike protein is significantly higher than its neutralization of the variant spike, such as the Delta variant (37). Our study can also confirm the reduction of neutralizing antibody protection against the Omicron variant spike. The salivary neutralization test in this study showed that the subjects’ mucosal neutralization against the Omicron spike RBD was abolished after the Pfizer BNT162b2 booster. It is interesting that neutralization in oral mucosal immunity is undetectable in individuals who received the BNT162b2 booster against either the reference spike RBD or the Omicron spike RBD (37). Yet, we detected neutralization in individuals who had the Pfizer booster but only responded to the reference spike RBD, not the Omicron variant spike RBD. Our results corroborate the finding that mucosal neutralizing immunity against Omicron is not elicited by the Pfizer/BioNTech (BNT162b2) or Moderna (mRNA-1273) mRNA vaccines, detected using pseudovirus neutralization assays that prevent virus entry into HEK 293T cells with the hACE2 enhancement (38).

## Conclusion

We provide an example of assessing a laboratory-developed surrogate virus neutralization test using supplies commonly available in a BSL-2 laboratory. This assay can be quickly deployed to meet urgent needs for oral mucosal neutralization immune evaluation in response to a particular pathogen threat. Our results indicate that the LDT sVNT can be used as a substitute for the commercial test. We found that oral mucosal virus neutralization is effective but strain-specific. Neutralization is lost when the Omicron variant spike protein carries multiple mutations that evade antibody binding.

## Data Availability

The original contributions presented in the study are included in the article/**Supplementary Material**. Further inquiries can be directed to the corresponding author.
