# Supplementary material for: Surrogate virus neutralization test to determine salivary neutralizing antibody inhibition of ACE2 and SARS-CoV-2 spike RBD complex
Source: Front Immunol. 2026 Jun 18;17:1803393. doi: 10.3389/fimmu.2026.1803393 (PMC13322917; doi:10.3389/fimmu.2026.1803393)

**Surrogate Virus Neutralization Test (sVNT) to determine salivary neutralizing antibody inhibition of ACE2 and SARS-CoV-2 Spike RBD complex**

David Forsman^a^, Abigail Smith^a^, Alex Pratt^b^, Paige Comerford^b^, Fiona Sparano^b^, Congyue Peng^a#^, Delphine Dean^a,c#^

^a^Department of Bioengineering, Clemson University, South Carolina, USA

^b^Department of Biological Science, Clemson University, South Carolina, USA

^c^Research and Education in Disease Diagnostics and Intervention, Clemson University, South Carolina, USA

#Address correspondence to Congyue Peng, [congyup@clemson.edu](mailto:congyup@clemson.edu) and Delphine Dean, finou@clemson.edu

**Supplementary tables and figures**

**Supplementary Table 1: Intra- and inter- assay variability of nAb, IgG, and IgA measurements in contrived saliva samples (negative saliva spiked with antibody standard).**

| **Measurement** | **Intra-assay coefficient of variation (*% CV*)** | **Inter-assay coefficient of variation (*% CV* )** |
| --- | --- | --- |
| nAb (U/mL) In-house | 7.85 | 9.67 |
| IgG (µg/mL) | 1.88 | 9.96 |
| IgA (µg/mL) | 2.10 | 33.08 |

**Supplementary Table 2: The Spearman correlation coefficient (Spearman’s *ρ* ) of the nAb measurement against the total IgA and IgG, the IgG against IgA.**

| **Measurement** | **Spearman’s *ρ*** |
| --- | --- |
| nAb vs. total of IgA and IgG | -0.123 |
| IgG vs. IgA | 0.285 |

**Supplementary Figure 1:** A line graph (left) showing the weekly fluctuation of neutralizing antibody titer determined by commercial GenScript sVNT assay for each participant. A bar graph (right) showing the overall weekly changes of the participants.

**Supplementary Figure 2:** A line graph (left) showing the weekly fluctuation of neutralizing antibody titer determined by in-house sVNT assay for each participant. A bar graph (right) showing the overall weekly changes of the participants.

**Supplementary Figure 3:** A line graph (left) showing the weekly fluctuation of IgG antibody titer for each participant. A bar graph (right) showing the overall weekly changes of the participants.

**Supplementary Figure 4:** A line graph (left) showing the weekly fluctuation of IgG antibody titer for each participant. A bar graph (right) showing the overall weekly changes of the participants.

Supplementary Figure 1


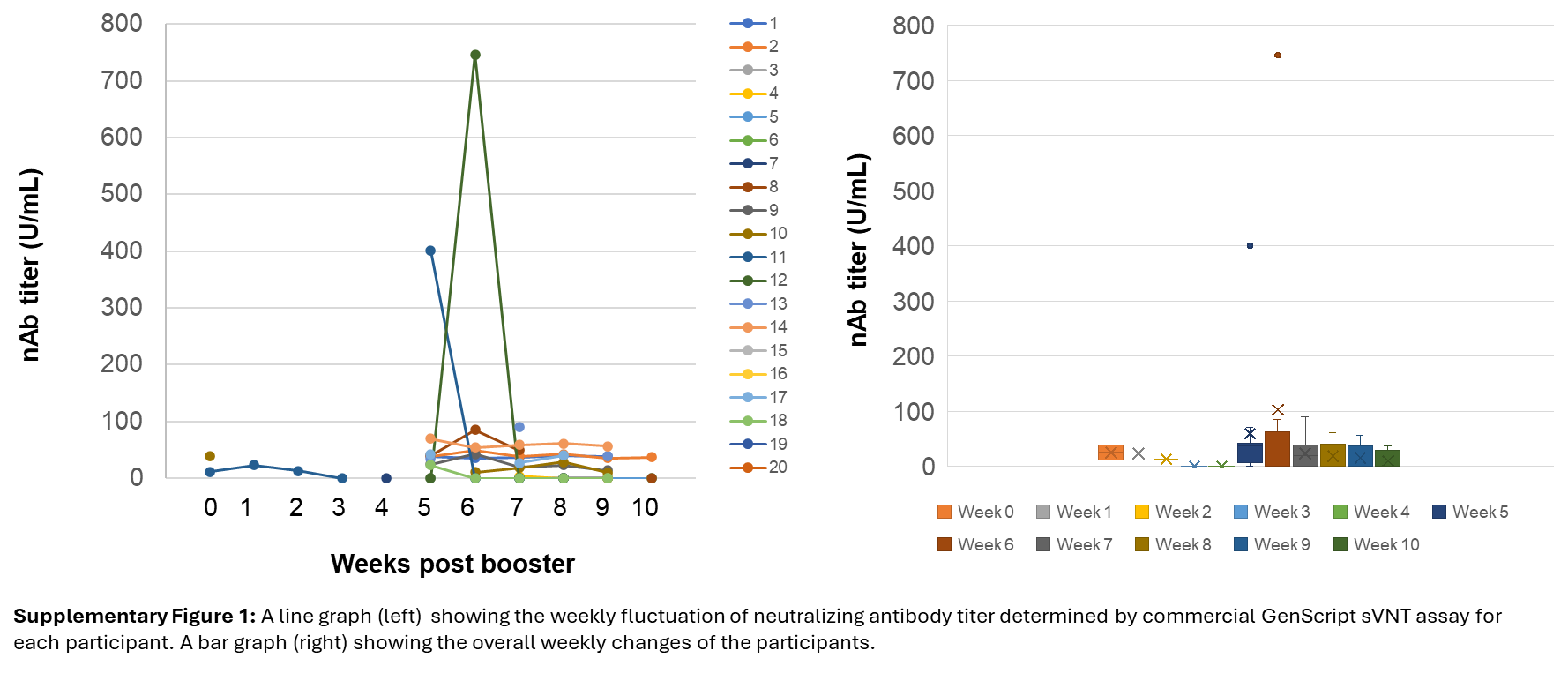


Supplementary Figure 2


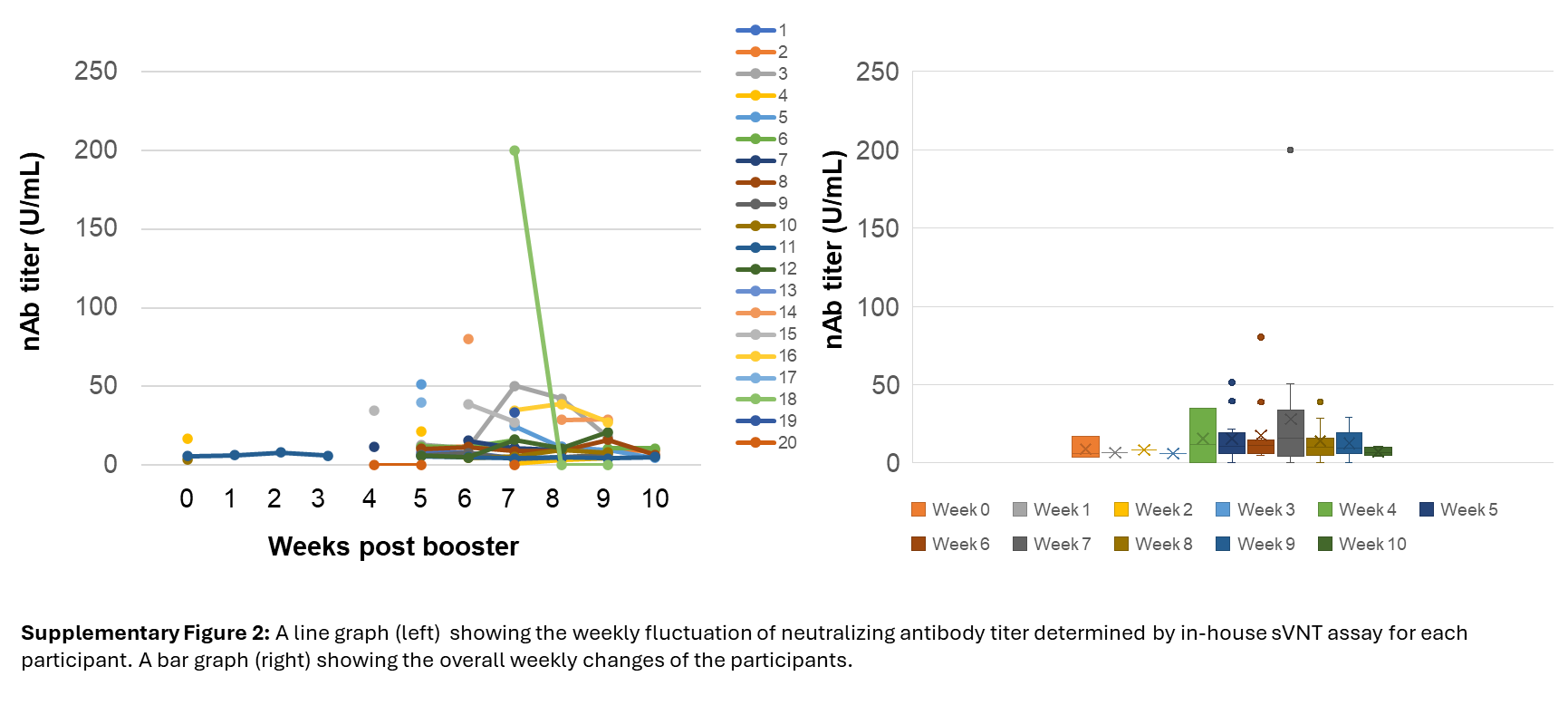


Supplementary Figure 3


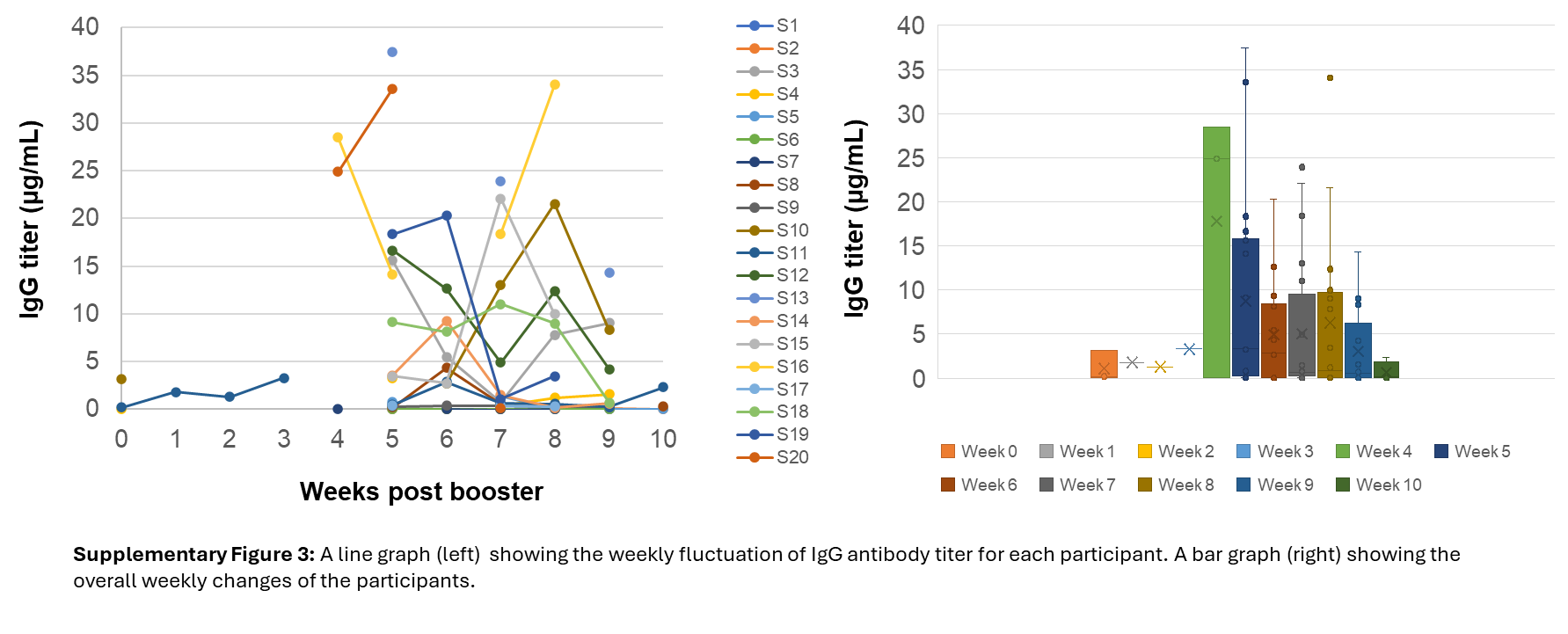


Supplementary Figure 4


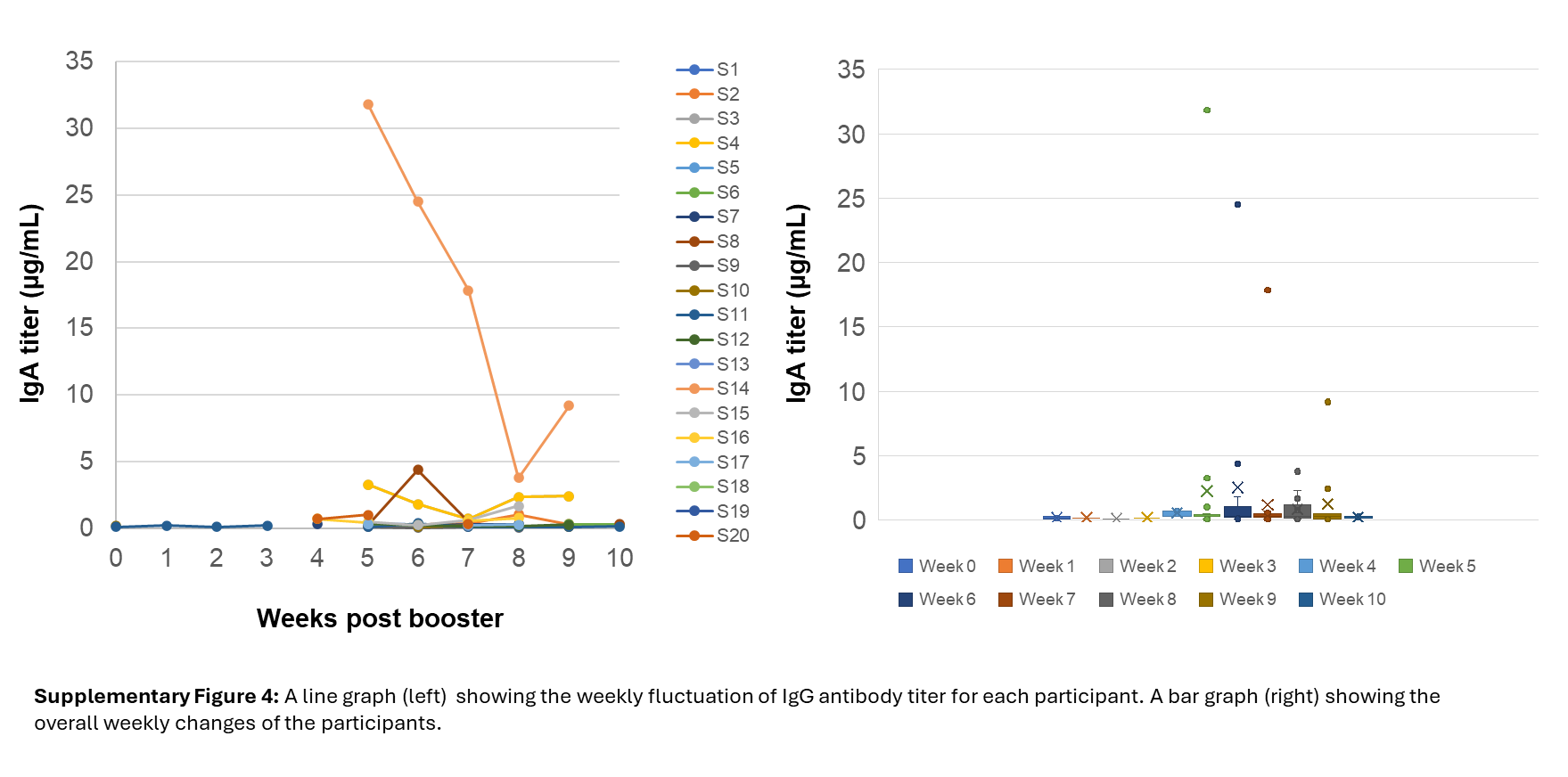

Supplement: Supplementary file 1 [file SupplementaryFile1.docx]
